# Dengue vector management using insecticide treated materials and targeted interventions on productive breeding-sites in Guatemala

**DOI:** 10.1186/1471-2458-12-931

**Published:** 2012-10-30

**Authors:** Nidia Rizzo, Rodrigo Gramajo, Maria Cabrera Escobar, Byron Arana, Axel Kroeger, Pablo Manrique-Saide, Max Petzold

**Affiliations:** 1 Centro de Estudios en Salud, Universidad del Valle de Guatemala, 18 Avenida 11-95 Zona 15, VH III, Guatemala, Guatemala; 2 Independent economic consultant, Johannesburg, South Africa; 3 Special Programme for Research and Training in Tropical Diseases, World Health Organization, (TDR,WHO), Av Appia, Geneva, 1211, Switzerland; 4 Liverpool School of Tropical Medicine, Pembroke Place, Liverpool, L3 5QA, UK; 5 Departamento de Zoología, Campus de Ciencias Biológicas y Agropecuarias, Universidad Autónoma de Yucatán, Carretera Mérida-Xmatkuil Km. 15.5, Mérida, C.P. 97315, Mexico; 6 Centre for Applied Biostatistics, Sahlgrenska Academy at University of Gothenburg, Medicinaregatan 16A, Gothenburg, 40530, Sweden

**Keywords:** Dengue vectors, Insecticide treated materials, Targeted interventions, Guatemala

## Abstract

**Background:**

In view of the epidemiological expansion of dengue worldwide and the availability of new tools and strategies particularly for controlling the primary dengue vector *Aedes aegypti*, an intervention study was set up to test the efficacy, cost and feasibility of a combined approach of insecticide treated materials (ITMs) alone and in combination with appropriate targeted interventions of the most productive vector breeding-sites.

**Methods:**

The study was conducted as a cluster randomized community trial using “reduction of the vector population” as the main outcome variable. The trial had two arms: 10 intervention clusters (neighborhoods) and 10 control clusters in the town of Poptun Guatemala. Activities included entomological assessments (characteristics of breeding-sites, pupal productivity, *Stegomyia* indices) at baseline, 6 weeks after the first intervention (coverage of window and exterior doorways made of PermaNet 2.0 netting, factory treated with deltamethrin at 55 mg/m^2^, and of 200 L drums with similar treated material) and 6 weeks after the second intervention (combination of treated materials and other suitable interventions targeting productive breeding-sites i.e larviciding with Temephos, elimination etc.). The second intervention took place 17 months after the first intervention. The insecticide residual activity and the insecticidal content were also studied at different intervals. Additionally, information about demographic characteristics, cost of the intervention, coverage of houses protected and satisfaction in the population with the interventions was collected.

**Results:**

At baseline (during the dry season) a variety of productive container types for *Aedes* pupae were identified: various container types holding >20 L, 200 L drums, washbasins and buckets (producing 83.7% of all pupae). After covering 100% of windows and exterior doorways and a small number of drums (where the commercial cover could be fixed) in 970 study households, tropical rains occurred in the area and lead to an increase of the vector population, more pronounced (but statistically not significant) in the control arm than in the intervention arm. In the second intervention (17 months later and six weeks after implementing the second intervention) the combined approach of ITMs and a combination of appropriate interventions against productive containers (Temephos in >200 L water drums, elimination of small discarded tins and bottles) lead to significant differences on reductions of the total number of pupae (P = 0.04) and the House index (P = 0.01) between intervention and control clusters, and to borderline differences on reductions of the Pupae per Person and Breteau indices (P = 0.05). The insecticide residual activity on treated curtains was high until month 18 but the chemical concentration showed a high variability. The cost per house protected with treated curtains and drum covers and targeting productive breeding-sites of the dengue vector was $ 5.31 USD. The acceptance of the measure was generally high, particularly in families who had experienced dengue.

**Conclusion:**

Even under difficult environmental conditions (open houses, tropical rainfall, challenging container types mainly in the peridomestic environment) the combination of insecticide treated curtains and to a less extent drum covers and interventions targeting the productive container types can reduce the dengue vector population significantly.

## Background

Dengue is the fastest expanding arboviral disease [1] presenting a serious public health challenge in endemic countries, particularly in South East Asia, Western Pacific Region, Latin America and now in the Middle East. As there is no drug or vaccine available, dengue vector control remains at present the only way of preventing disease transmission and reducing the burden of disease.

There are a number of chemical or non-chemical vector control tools available [2] which are effective if correctly applied [3,4]. However, their efficacy is limited because they are either not applied in an efficient way by vector control services [5,6], applied in vertical programs without involvement of communities and other partners [7-11] or over-stress ULV (Ultra Low Volume) fogging [12] as one of the most prominent “technocratic” approaches [13]. This is why, user friendly and cost-effective, new tools/interventions for dengue vector management such as targeting only the most productive (for adult vector development) water container types [14] and insecticide treated materials (ITMs) [15-17] are being tested to complement and enhance current dengue vector control.

Here we report the efficacy, acceptance and costs of an intervention with a combination of insecticide treated materials (Long Lasting Netting Materials, LNs) deployed as window and external door curtains plus covers of 200 L drums and targeting productive *Aedes aegypti* breeding-sites over a period of 18 months in the locality of Poptun Guatemala (from March 2009 to October 2010).

## Methods

### The study site

Poptun, a small town with approximately 35,000 inhabitants in El Petén in Guatemala, was selected on purpose because of the increasing numbers of dengue cases in recent years. Dengue is perceived to be an important public health problem with 87 confirmed cases in 2010 (Laboratorio Nacional de Salud; unpublished report). The town is situated in a tropical humid area with an average annual temperature of 26°C, an average relative humidity of 80% and annual rainfall of 1,700 mm with two distinguishable seasons: a dry season from November to the beginning of May and a wet season from May to October. Most houses, mainly one-storey buildings, have a small patio with some vegetation. They have electricity and half of them have piped drinking water, but usually only for some hours a day; therefore, the population stores water in large and small containers (see below). Houses were generally made of a combination of wood and concrete, mostly with roofs made of corrugated metal treated with zinc; they had on average three windows, the majority of them uncovered and unprotected, and had two exit doors, often open for better ventilation, one at the front (facing to the street) and one at the backyard. During the dry season, most people remain outside their houses during the afternoon until dusk.

### Design of the cluster randomized community trial including sample size

As we intended to test the efficacy of an area intervention (see later: interventions 1 and 2), a cluster randomized trial design was adopted using as the main outcome variable “reduction of vector density” measured through pupal indices. A cluster was defined as a neighborhood with approximately 100 adjacent houses and a “buffer zone” with untreated houses between clusters of around 50 to 100 metres in order to minimize the “spillover effect” of *Aedes* mosquitoes flying from control to intervention clusters [15]. Experience from previous studies has shown that 20 clusters with a total of 2000 households were feasible to cover with the different study instruments [15]. To calculate the minimal cross-sectional difference to be detected with a power of 80% at a significance level of 5% given this sample size, pupae per person index (PPI) levels and variability on study cluster level were applied from a previous study [18]. Assuming a normal distribution of PPI over clusters and a standard deviation of 0.1 the minimal difference was found to be 0.12.

### Cluster selection

**Figure 1:**
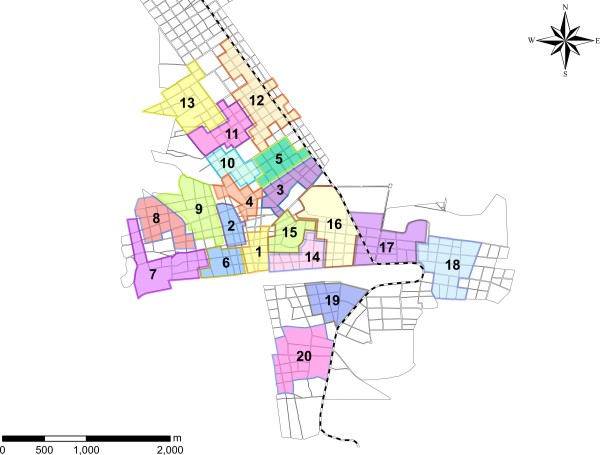
**Map of blocks of houses (“cuadras”) of the town of Poptun, Guatemala, with the 20 study clusters (Pairs of clusters: 1 and 4, 8 and 13, 3 and 18,14 and 16, 6 and 7, 11 and 17,10 and 15, 19 and 20, 2 and 12, 9 and 5)** .

**Figure 2:**
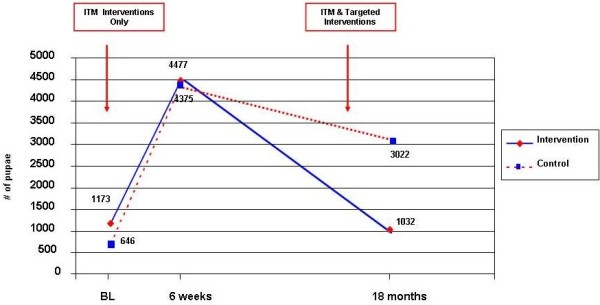
**Efficacy of the first and second intervention in reducing vector densities (using total number of pupae per cluster; p values in the text)** .

The town was divided into 20 clusters surrounded by the buffer zones (see map, Figure 1). After the entomological baseline survey (which followed the written consent by heads of household; see ethical issues) pairs of clusters with similar characteristics in terms of pupal indices were identified. Subsequently, random allocation within pairs of clusters to intervention and control group was done by throwing a coin (10 intervention clusters and 10 control clusters). From an initial number of 2,357 houses, only 1,835 were followed through the whole study period of 18 months and were included in the analysis; the other houses either belonged to the buffer zones or dropped out (see Figure 2).

### The first intervention to be tested: LNs (long lasting netting materials) deployed as curtains and 200 L drum covers

A total of 3,079 readymade PermaNet® 2.0 curtains (deltamethrin coated on polyester netting at 55 ± 13.75 mg/m^2^ with 75 denier) with a standard size (1.0 × 1.7 m for windows and 1.2 × 2.2 m for outside doors) and 298 drum covers (75 cm diameter) with an inner circle of PermaNet 2.0 and an elastic band with an outer circle of stronger textile (treated phtalogen blue woven dumuria fabric, 150 denier, 100% polyester) were donated by Vestergaard-Frandsen company in Lausanne, Switzerland. People in the intervention clusters had been asked beforehand to choose a color (white, blue, pink, green). The curtains were fixed with a string and two nails by the study team. PermaNet curtains were fixed beneath existing curtains in some houses. Through the excellent community participation all houses in intervention clusters accepted their windows and outside doors covered by curtains. The National vector control services continued with their routine programme and treated at the same time all water containers in three control clusters and three intervention clusters with Temephos 1% (1 ppm concentration applied every 3 months).

### The second intervention to be tested: LN curtains/drum covers and targeted intervention in productive containers

A second intervention was implemented 17 months after the baseline survey and at the beginning of the rainy season. It included the replacement of the existing IT window curtains (as many of the original ones were damaged), covering 298 drums with new Permanet covers (also because of damaged material) and treating the productive container types previously identified (washbasins and “other” large water containers with >20 L -including wells, water deposits for cattle, water tanks or play basins for children with Temephos 1% at 1 ppm) and emptying buckets and eliminating disposable items. At the same time the National Vector Control Program continued their routine programme and treated the water containers in three control clusters and three intervention clusters with Temephos (see above).

### Entomological assessment: pupal productivity surveys and larval surveys

For the pupal productivity surveys a SOP (Standard Operational Procedure) was developed [19]. All pupae in the breeding sites were counted and examined for species and sex (after emergence of adult mosquitoes) in the Laboratory. The number of pupae was used as a proxy to the number of adult mosquitoes [20,21]. The primary outcome variables for the intervention were “total production of *Aedes* pupae” and PPI (Pupae per Person Index which is the ratio between pupae and persons living in each cluster). Additionally, the *Stegomyia* indices i.e. House, Container and Breateu indices were calculated. Pupal demographic surveys were conducted by experienced health inspectors from the Ministry of Health and from the University del Valle de Guatemala. The surveys were conducted at baseline, six weeks after the first intervention and six weeks after the second intervention that was implemented 17 months after baseline.

### Data management and statistical analysis of entomological indices

Data entry and cleaning using ACCESS was done by the computer center at the Universidad del Valle de Guatemala and data analysis at the Nordic School of Public Health in Gothenburg, Sweden. The analysis of entomological indices was done at cluster level e.g. PPI (pupae per person index) was calculated per cluster and differences between intervention and control clusters were then tested using t-test. Differences (change) between intervention and control clusters over time were tested as the average of the within-cluster change from baseline to the two different follow up surveys (follow up survey value minus baseline value).

### Analysis of coverage and people’s acceptance

The correct use of treated curtains and drum covers was checked by the research team every third month (in total seven check visits during the project period) in the total number of intervened houses. Additionally, a household survey with a formal questionnaire was conducted in month 15 by trained interviewers in 370 houses to determine user’s satisfaction and knowledge, attitudes and practices (KAP) about dengue among the intervention group. This was complemented by five Focus Group Discussions (FGDs) conducted by a medical anthropologist together with an assistant. ITMs user’s acceptance survey focused on what users liked or disliked about windows and/or door curtains and drum covers, color and pattern preferences and type of material.

### Bioassays for determining bioavailability of the insecticide

Standard World Health Organization cone bioassays [22] were performed on different samples of PermaNet® 2.0 curtains in two laboratories: a) in Tapachula, Mexico (Insecticide Evaluation Laboratory of the Centro Regional de Investigación en Salud Pública-CRISP) after 9 months of use. Groups of five susceptible, non-blood fed, 2-day old *Aedes aegypti* (New Orleans strain) were exposed to netting materials (25 cm x 25 cm) for 3 minutes, under WHO cones and held for 24 h in paper cups with access to a 10% sucrose solution. Bioassays were also performed for two new and non-exposed net samples. In addition, groups of resistant and wild *Ae. aegypti* (Progreso and Tapachula strains respectively) female mosquitoes were exposed to the netting material. As control for each sample a total of 30 mosquitoes were exposed to new untreated white nets. Mortality was recorded after 24 h. Data were pooled and percent-corrected mortality was calculated and corrected when the mortality in control replicates was >5 and <20% using Abbott’s formula [23]; b) In the same way bioassays were carried out at the Liverpool School of Tropical Medicine, UK, under standard lab conditions of 25°C and 75% RH using susceptible *Aedes aegypti* strains ( groups of five susceptible, non-blood fed, 2-day old *Aedes aegypti* and 30 controls exposed to untreated nets). The 24 vector mortality after the 3-minutes exposure was determined and corrected with Abbot’s formula; the Liverpool samples included PermaNet 2.0 materials used in Poptun for 3, 12 and 18 months, 10 replicates for each sample.

### Chemical analysis of netting materials

All samples were analyzed by high-performance liquid chromatography (HPLC) at the Liverpool School of Tropical Medicine as follows. Five circular pieces of net (7 cm^2^) were cut randomly from each curtain to minimize variation and then cut into small pieces into a glass vial; and afterwards subjected to acetone extraction by vortexing 3 x with 3 ml acetone, combined and evaporated to dryness under a stream of nitrogen. The deltamethrin residue was recovered in 1 ml acetonitrile and filtered with a 17 mm PTFE 0.2 μm syringe filter (Elkay, Basingstoke, Hampshire, UK) before analysis. HPLC analyses were performed by injection of 20 μl aliquots on a reverse-phase Dionex Acclaim C18 column (120 Å, 250 x 4.6 mm, 5 μ, Dionex, Camberley, UK). A mobile phase of methanol/water 90:10 was used at a flow rate of 1 mL min-1. The quantities of insecticides were calculated from standard curves established by known concentrations of authenticated deltamethrin peaks detected using an Ultimate 3000 UV detector and analyzed with Dionex Chromeleon software. Additional samples were analyzed at Vestergaard-Frandsen laboratories in Hanoi, Vietnam to check on the consistency with the Liverpool tests. They extracted the samples in a mixture of n-hexane and 1,4 dioxane. The deltamethrin content was determined by normal phase high performance liquid chromatography (HPLC) using dibutyl phthalate as internal standard and detection at 236 nm.

### Cost analysis of the intervention with treated curtains and drum covers

The cost analysis included only those costs that the vector control services or other agencies would have to cover if they intend to do a dengue vector control program using insecticide treated curtains and targeted intervention. The costs recorded during the interventions were: staff costs for 30 working days (to cover all households in the intervention clusters), transport costs for staff as well as depreciation of the vehicle in use, minor equipment such as hammers and nails and consumables (mainly food for the staff). The price of curtains was difficult to establish as the product is not yet on sale and the price depends on the numbers ordered. The life expectancy of curtains was estimated to be 2,5 years. The exchange rate of Quetzal (local currency) to US Dollar was in November 2008 = Q7.63 according to the Central Bank of Guatemala. Both “total costs” and “cost per house protected” were estimated.

### Ethical aspects

The study protocol was reviewed and approved by both; the UVG ethics committee and the ethics review committee at WHO. A signed consent form was obtained from all heads of households before the start of the study.

## Results

### Characteristics of the study population

The study population consisted of 20 neighborhoods (clusters) with 1,835 households and a population of 8,875 inhabitants: 865 households (4,111 people) within the 10 control clusters and 970 households (4,764 people) in the 10 intervention clusters (Figure 1, Table 1*).*

**Table 1:** Baseline information of the study population at Poptun Guatemala

	**Control**	**Intervention**	**Total**
No. of clusters	10	10	20
No. of households	865	970	1,835*
No. of inhabitants	4,111	4,764	8,875
No. of people per house	4.75	4.91	4.84

#### Vector breeding at baseline during the dry season

At baseline during the dry season a total of 8,558 water holding containers were recorded, most of them (79.2%) located outdoors and only 20.8% indoors; 134 of them (1.6%) were positive for *Aedes* pupae producing a total of 1,783 pupae at the time of inspection. A total of 15 different container types were identified; four of these container types (representing 52.0% of all water holding containers) were responsible for 87.3% pupal (and consequently adult *Aedes*) production: water containers with >20 L of water -including wells, water deposits for cattle, water tanks and play basins for children-(producing 33.7% of all pupae), buckets (22.4%), drums (15.8%) and large wash basins (15.4%).

The *Stegomyia* indices (House Index, HI = % of houses infested with *Aedes* immature stages, Container Index, CI = % of infested water holding containers, Breteau Index, BI = number of infested containers per 100 households) were fairly the same in intervention and in control clusters and ranged from low to intermediate: HI roughly 10%, CI 3%, BI 13).

### The first intervention (ITMs) during the dry season and its acceptance

The first intervention consisted of installing 3,079 PermaNet 2.0 curtains in all 970 study households of the intervention clusters (on average three curtains per house covering 100% of windows and outside doors). Additionally 298 drums were protected with treated covers. The low number of drum covers distributed was because only around 25% of the households had containers where the commercially produced covers could be fixed; i.e. there were a large number of unprotected water containers. meaning that the effect of the first intervention was basically due to the treated curtains.

Observation and repeated household surveys showed that during the study period, more than 96% of the households used the treated curtains and drum covers. However, only around 83% of the door curtains and 74% of the window curtains were correctly placed at the time of observation, since some families used to tie the curtains during daytime for better ventilation or did not keep the drum covers fixed with the elastic band. Approximately 10% of both curtains and container covers were found damaged (broken or with holes) at the time of the supervision visits. As mentioned before the National vector control program treated the water containers in three control clusters and three intervention clusters with Temephos.

### Vector breeding 6 weeks after the first intervention during the wet season

After the intervention heavy rainfalls occurred in the area and vector breeding changed dramatically. The number of water-holding containers increased in the study area from 8,558 to 8,965. Most of the water containers were outdoors both in the intervention clusters (84.3%) and in the control clusters (85.8%). The total number of pupae collected increased to 8,885 in the study households. The increase was more pronounced in the control area (from 646 at baseline to 4,375 at follow up) than in the intervention area (from 1,137 to 4,477) but the difference was not statistically significant (P = 0.60). The PPI (pupae per person index) was higher in the control arm compared to the intervention arm, but the difference was not statistically significant (P = 0.80, Table 2).

**Table 2:** Change in PPI (as a proxy measure for vector densities) from baseline to 6 weeks and 18-month surveys, respectively

	**Mean change intervention clusters (95% CI)**	**Mean change control clusters (95% CI)**	**P-value**
After 1^st^ intervention	0.84 (0.61 to 1.07)	0.79 (0.45 to 1.14)	0.80
After 2^nd^ intervention	−0.01(-0.26 to 0.24)	0.52 (-0.01 to 1.05)	0.05

P-value given for difference in change between intervention and control clusters.

In the intervention clusters the productive container types were the same as before plus discarded car tires producing together 72.1% of all pupae. The *Stegomyia* indices showed increases; particularly in the control clusters in comparison to the intervention clusters but the differences were not statistically significant (not shown in a table because being of secondary importance).

### The second intervention during the wet season and its acceptance

In the second intervention, all windows and outside doors at the intervention households were covered with Permanet2 curtains (on average three curtains per household) reaching 100% coverage and the 298 drums were covered with the above described water container covers; additionally the productive container types were eliminated or treated (see methods).

The assessment of people’s acceptance showed that the majority was happy to receive the materials (particularly curtains) free of cost, but many were not satisfied with the quality of the curtains and would prefer materials of a better quality. About 50% mentioned that they preferred colored curtains, and only 15% preferred white curtains. The greatest perceived benefit was that users could clearly see the dead insects beneath the curtains, which reassured them of the protective effect Some households reported skin and eye reactions in children who stayed for a prolonged period close to the curtains. The most frequently reported inconvenience was that the curtains hindered the circulation of air. Overall, the perceived benefit of using treated netting materials was greater in the households where a case of dengue had previously been reported.

### Vector breeding 6 weeks after the second intervention

In the subsequent entomological follow up survey the following was encountered. The total number of water-holding containers was 9,120: 5,037 in the intervention clusters and 4,084 in the control clusters. The large majority of containers were found outdoors (85.3%) with no difference between intervention and control arms. The pupal production was much lower compared to the previous pupal productivity survey with 4,054 pupae in both study arms together. There was a significant difference in the reduction of number of pupae (P = 0.04; Figure 2) and a borderline significant reduction of the PPI (pupae per person index) between intervention and control arms (P = 0.05; Table 2).

In the intervention area washbasins and large “other” containers (including wells, water deposits for cattle, water tanks or play basins for children) remained important for pupal production (together 32.5% of all pupae), but also in other container types where the intervention was less effective: buckets (17.0% of pupae production) and small and medium sized bowls (20.5%). In the control areas washbasins (23.9% of pupae production), tires (11.5% production) and drums (7.1% production) remained important but were complemented by “natural breeding places” (such as coconut shells and plants, 16.9% production) and buckets (11.2% production), which had already been important in the baseline survey. In both intervention and control arms, the majority of productive containers were found outdoors (81.1% and 78.4%, respectively).

Regarding the *Stegomyia* indices, there was a significant difference on the reduction of HI (P = 0.01), and a borderline statistical reduction of BI (p = 0.05) between intervention and control clusters. No significant difference was observed on the CI (P = 0.10).

### Insecticide residual activity in treated curtains and drum covers

According to bioassays results in Tapachula, Mexico (Table 3), the residual activity of insecticide on the curtains was high after nine months of use, exposure to sunshine and washing (Table 3). Both susceptible New Orleans strains and wild Tapachula strains of *Aedes aegypti* exposed to new and 9 months used IT curtains had Abbot corrected mortalities >80%; only the resistant Progreso strain showed low 24 h mortality. Bioassays tests done in Liverpool (Table 4) after 3, 12 and 18 months showed a consistently high 24 h mortality.

**Table 3:** Insecticide bioavailability of PermaNet® 2.0 curtains after 9 months of use in Poptun, Guatemala

**No. Aedes females/replicate**	**Observations**	***Aedes *strain**	Abbot corrected 24 h mortality*
			**(%)**
5 per group	Bedroom, Always shade, No wash	New Orleans (Susceptible)	100.0
		Progreso (Resistant)	27.5
		Tapachula (Wild)	100.0
5 per group	Bedroom, Full sun, Washed 3 times, Soaked with cold water and bar soap	New Orleans (Susceptible)	88.1
		Progreso (Resistant)	5.8
		Tapachula (Wild)	89.2
5 per group	Bedroom, Full sun, 2 washes, Scrubbed with cold water and bar soap	New Orleans (Susceptible)	92.3
		Progreso (Resistant)	0.8
		Tapachula (Wild)	91.7
5 per group	Bedroom, Full sun, No wash	New Orleans (Susceptible)	92.6
		Progreso (Resistant)	0.8
		Tapachula (Wild)	98.3
5 per group	Bedroom, Full sun, No wash	New Orleans (Susceptible)	81.9
		Progreso (Resistant)	9.2
		Tapachula (Wild)	88.3
5 per group	Unused	New Orleans (Susceptible)	100.0
		Progreso (Resistant)	25.8
		Tapachula (Wild)	100.0
5 per group	Unused	New Orleans (Susceptible)	98.1
		Progreso (Resistant)	4.1
		Tapachula (Wild)	100.0

* 30 female *Aedes* mosquito controls exposed to un-treated nets for each experiment (see text).

**Table 4:** Insecticide bioavailability and chemical residuals of PermaNet 2.0 curtains and drum covers used in Poptun, Guatemala, over a period of 18 months /March 2009 to October 2010

**Type of ITM and months of use**	**No. Aedes females/replicate**	**Abbot corrected 24 h mortality (%)**	**No. ITM samples**	**HPLC (mg/m**^**2**^ **± SD)**
3 months (Curtains)	5	100	10	54.7 (30.1)
12 months (curtains)	5	100	10	53.9 (20.7)
18 months (curtains)	5	100	10	64.9 (16.6)
3 months (drum covers)	5	100	5	--
18 months (drum covers)	5	85.3	10	--

(The activity of deltamethrin was assessed by the WHO standard cone test. Batches of females of the Liverpool strain (susceptible) were exposed for 3 minutes to different samples of the netting materials and mortality rate after 24 h was recorded. The chemical analysis of the residuals was performed by HPLC. Both assays were carried out at the Liverpool School of Tropical Medicine, UK). Great Britain.

* 30 female *Aedes* mosquito controls exposed to un-treated nets for each experiment (see text).

### Chemical analysis of ITs

The analysis of chemical residuals by HPLC showed high average values but also a high variation of results in different replicates after 3, 12 and 18 months of use (Table 4) but the original concentration of deltamethrin (55 ± 13.75 mg/m^2^ according to the manufacturer) has not been tested The comparative analysis done by another laboratory (Vietnam) produced similar results. High deltamethrin retention was observed in five curtains tested after 3 months (66.6 mg/m^2^ +/− 19.4 SD), 6 months (47.4 mg/m^2^ +/−23.7 SD), 12 months (53.9 mg/m^2^) and 18 months (64.9 mg/m^2^) of use. The retention of insecticide in five drum covers was also considerable but with a large variation among samples after 3 months of use with 28.6 mg/m^2^ (+/−21.3 SD) and after 6 months of use with 39.1 +/− 21.4 mg/m^2^ in the central netting section.

### Cost analysis of the intervention

The National vector control program would have to pay in total an equivalent of $ 5,842 US Dollars for covering all intervention houses in Poptun ($ 5.31 US Dollars per house protected; Table 5). Most of the cost was due to staff salaries (69% of the total) and to materials/consumables (20.0%) and less to transport costs (11%). The cost of the curtains and water container covers was not included as they are highly variable and dependent on the size of the purchase, the import duties and negotiation with the provider.

**Table 5:** Cost components of the combined intervention (ITN on window/doors and water container covers plus targeting productive breeding-sites) and direct costs to the vector control program

**Costs per work cycle**		
Recurrent costs	Quetzales	USD $ 2010
Staff Costs	31,811.00	$4,018.78
Consumables	9,286.20	$1,155.00
Transport costs	3,738.28	$464.96
Capital costs		
Vehicles	134.12	$16.68
Minor equipment	2,238.93	$186.58
Total cost per cycle	47,208.53	$5,842.00
Total cost per house	42.92	$5.31

## Discussion

### Efficacy of treated curtains and drum covers

The impact of commercially treated curtains and a relatively small number of drum covers on vector abundance was less dramatic when compared to previous cluster randomized trials [15,16] although the level of coverage, recognized to have important implications for the effectiveness of interventions, was high in Poptun and similar to these preceding studies. However, a number of factors may have reduced the efficacy of insecticide treated materials: a) The “spill-over effect” affecting mosquito densities in intervention but also in controls clusters, [15]; b) Vector control activities by the Ministry of Health both in intervention and control clusters may have confounded the study results by also reducing vector densities in the control clusters; c) The majority of large productive water containers remained un-covered as the number of water holding drums for which the commercially produced covers were designed was small (particularly during the wet period) compared to the other large water containers with different shapes and sizes; therefore in the first intervention during the dry period the protection of drums with Permanet 2 covers turned out to be insufficient as the torrential rains filled many other large and small containers during the wet period; d) Recent evidence shows that doing the baseline pupal productivity survey during the dry season (this was the case in the first part of our study) leads to an underestimation of productive container types in the wet season so that interventions targeting the most productive container types may miss some of them [24]. e) Vector breeding and interaction with humans seems to occur in Poptun mainly in the peridomestic environment; and vectors are not required to move frequently through windows and doors between the intradomestic and extradomestic spaces as the main breeding places are mostly outdoors where people work and live.

### Efficacy of the combined approach: treated materials and targeted interventions

The combination of treated curtains and drum covers with targeted interventions in productive container types was successful in reducing the number of *Aedes* pupae and consequently of adult dengue vectors. However, the effect could have been even better if the Temephos treated large water containers would have had a longer lasting efficacy: many of them were re-infested and produced pupae (and thus adult mosquitoes) 6 weeks after the intervention. High water turnover and/or Temephos resistance may be the explanation. Likewise the management of buckets by the community was insufficient leading to considerable pupal production in this container type. If the reduction of the vector density achieved by our intervention is sufficient for reducing or interrupting dengue transmission is unclear. The observation that the pupae per person index (PPI) in the wet season (follow up after the 2^nd^ intervention) came down to the value of the baseline PPI in the dry season when little or no dengue cases are reported may indicate that the limitation of adult production was sufficient for substantially reducing transmission and disease. However, this cannot be proven in this kind of study where the viral transmission to any inhabitant of Poptun may take place in control as well as in intervention clusters.

### Bioassays and chemical analysis

Bioassays to determine the availability of the insecticide on the surface of the fiber (insecticide residual activity) and chemical analysis of the deltamethrin content in the coating substance on the polyester fibers showed satisfactory results for at least 18 months of exposure in curtains and 6 months in water container covers (no further testing; Table 4). Some variation in bioassay results seem to be due to the exposure of treated curtains to sunshine and washes (analysis in Mexico) but the 24 hours mortality was in all samples above the 80% threshold. The high value of chemical concentration in one sample of the Liverpool analysis (64 +/−16 mg/m^2^) as well as the considerable variability in chemical content was probably due to the variation of the original chemical concentration in the treated curtains supplied but also as far as low concentrations are concerned, due to human behavior (frequent washing of nets) and exposure to sun light. The need for using high quality products shall be stressed and emphasis should be placed on quality assurance, both at factory level production to reduce variability in chemical content and at the procurement stage by a pre- and post-inspection quality control.

### Cost

The cost of $5.31 US Dollars plus (variable) cost for ITMs per house protected were in the range of an insecticide treated bed net for malaria control [25]. However, staff costs are part of the normal budget of control services as well as materials (consumables) such as stationary as well as transport costs. There is room for cost savings when the interventions are integrated into the routine vector control program.

## Conclusion

Even under difficult environmental conditions (open houses, tropical rainfall, challenging container types mainly in the peridomestic environment) the combination of ITMs as curtains and interventions targeting the productive container types can reduce the dengue vector populations significantly.

## Competing interests

The authors declare that they have no competing interests.

## Authors’ contributions

PM, AK, NR, BA designed the study. NR and RG were responsible for the development of the study in the field. All authors shared in the interpretation of data and writing the manuscript, and all approved the final manuscript. PMS is the guarantor of this paper.

## Pre-publication history

The pre-publication history for this paper can be accessed here:

http://www.biomedcentral.com/1471-2458/12/931/prepub
